# Lung cancer biopsies: Comparison between simple 22G, 22G upgraded and 21G needle for EBUS-TBNA

**DOI:** 10.7150/jca.48691

**Published:** 2020-09-14

**Authors:** Paul Zarogoulidis, Dimitris Petridis, Konstantinos Sapalidis, Kosmas Tsakiridis, Sofia Baka, Anastasios Vagionas, Wolfgang Hohenforst-Schmidt, Lutz Freitag, Haidong Huang, Chong Bai, Dimitris Drougas, Vasiliki Theofilatou, Konstantinos Romanidis, Eleni-Isidora Perdikouri, Savas Petanidis, Bojan Zaric, Tomi Kovacevic, Vladimir Stojsic, Tatjana Sarcev, Daliborka Bursac, Biljana Kukic, Branislav Perin, Nikolaos Courcoutsakis, Evagelia Athanasiou, Dimitrios Hatzibougias, Konstantinos Drevelegas, Ioannis Boukovinas, Maria Kosmidou, Christoforos Kosmidis

**Affiliations:** 1 3rd University General Hospital, “AHEPA” University Hospital, Thessaloniki, Greece.; 2 Department of Food Technology, School of Food Technology and Nutrition, Alexander Technological Educational Institute, Thessaloniki, Greece.; 3 Thoracic Surgery Department, “Interbalkan” European Medical Center, Thessaloniki, Greece.; 4 Oncology Department, “Interbalkan” European Medical Center, Thessaloniki, Greece.; 5 Oncology Department, (NHS) Genaral Hospital of Kavala, Kavala, Greece.; 6 Sana Clinic Group Franken, Department of Cardiology/Pulmonology/Intensive Care/Nephrology, “Hof” Clinics, University of Erlangen, Hof, Germany.; 7 Department of Pulmonology, University Hospital Zurich, Rämistrasse 100, 8091, Zurich Switzerland.; 8 Department of Respiratory Medicine, Changhai Hospital of Second Military Medical University, Shanghai, China.; 9 Scientigraphy Department, “Bioclinic” Private Laboratory, Thessaloniki, Greece.; 10 Second Department of Surgery, General University Hospital of Alexandroupolis, Medical School, Democritus University of Thrace, Alexandroupolis, Greece.; 11 Oncology Department, General Hospital of Volos, Volos, Greece.; 12 Department of Pulmonology, I.M. Sechenov First Moscow State Medical University, Moscow, Russian Federation.; 13 Faculty of Medicine, University of Novi Sad, Institute for Pulmonary Diseases of Vojvodina, Novi Sad, Serbia.; 14 Radiology Department, Democritus University of Thrace, Alexandroupolis, Greece.; 15 Private Pathology Laboratory, “Microdiagnostics”, Thessaloniki, Greece.; 16 Radiology Department, “Euromedica” Private Radiology Laboratory, Thessaloniki, Greece.; 17 Oncology Department, “Bioclinic” Private Hospital, Thessaloniki, Greece.; 18 Internal Medicine Department, University of Ioannina, Ioannina, Greece.

**Keywords:** Mediglobe, Olympus, EBUS, lung cancer, bronchoscopy, cell blocks, lymphnodes, NSCLC

## Abstract

**Introduction:** Novel technologies are currently used for lung cancer diagnosis. EBUS-TBNA 22G is considered one of the most important tools. However; there are still issues with the sample size.**Patients and Methods:** 223 patients underwent EBUS-TBNA with a 21G Olympus needle, 22GUS Mediglobe and 22GUB Mediglobe. In order to evaluate the efficiency of 22GUB novel needle design. In order to evaluate the sample size of each needle, we constructed cell blocks and measured the different number of slices from each biopsy site.

**Results:** The 22GUB novel needle had similar and larger number of slices from each biopsy site compared to 21G needle.

**Discussion:** Firstly as a novel methodology we used the number of slices from the constructed cell blocks in order to evaluate the sample size. Secondly, we should seek novel needle designs and not only concentrate on the volume of the sample size.

## Introduction

Currently we have novel equipment for lung cancer diagnosis and staging. We use bronchoscopy, endobronchial ultrasound radial and convex, electromagnetic navigation, archimedes virtual bronchoscopy, CT guided biopsy and ultrasound guided biopsy 1-3. Medical thoracoscopy is also used as a technique 4-6. Positron emission tomography (PET-CT) is an advanced imaging technique that is used as a surrogate in diagnosis and staging 7. Endobronchial ultrasound with convex probe and PET-CT combined provide in several lung cancer cases the staging of the disease. In advanced stage disease molecular biomarkers such epidermal growth factor (EGFR), anaplastic lymphoma kinase (ALK), proto-oncogene B-Raf (BRAF), proto-oncogene tyrosine-protein kinase (ROS1) and programmed death-ligand 1 (PD-L1), provide us with useful information regarding the treatment of the patient 8-11. PD-L1 can be efficiently assessed with cell-blocks 11-13. However; an important issue still remains for the sample size obtained from the EBUS-TBNA system. Currently 22G needles are mostly being used and afterwards 21G needles. We have different 22G and 21G needle designs; however, few studies have been made comparing the samples between different needle types. Moreover; we can use 19G needles with the EBUS-TBNA convex system, however; this is not a common practice especially for lung cancer staging where the lymphnodes can be ≤1 cm in diameter. It is already known that EBUS-TBNA uses 19G needles, 22G needles and 21G needles. 19G needle having the largest diameter and it is only tissue if of course the lesion has no necrosis. The two techniques are totally different, EBUS-TBNA is an endoscopic technique and Video Assisted Thoracic Surgery -VATS is a surgical technique and of course in VATS the surgeon can take a larger piece of tissue than the operator with the EBUS-TBNA 14, 15. Another issue is how to measure the sample size, if it is enough for molecular analysis, even for next generation sequencing (NGS) where just a small tissue slice is enough for molecular analysis 10. In order to measure the sample size we decided to measure the number of glass slices produced from the cell blocks obtained which we will discuss in detail in the patients and methods section. In the current study, we wanted to evaluate whether the 22G needle is as efficient as a 21G needle and how the different needle design can affect the diagnostic outcome. This study is based on the practice of EBUS-TBNA 22G and 21G where there are false negative for both needle sizes.

## Patients and Methods

**Figure 1:**
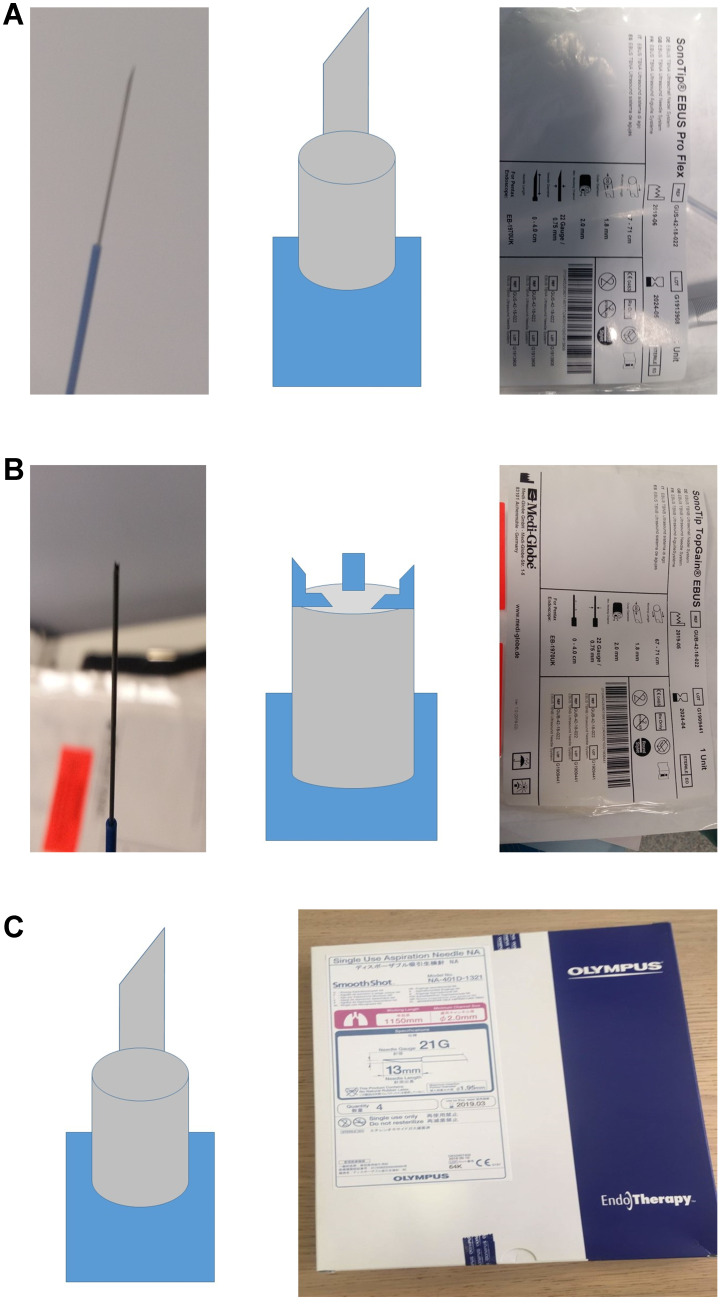
** A.** 22GUS needle Mediglobe^®^ (called simple needle 22G). **B.** 22GUB Mediglobe^®^ (called modified needle 22G). The tip has four small anchors. **C.** 21GUS needle Olympus^®^.

Two hundred and twenty three patients were enrolled in the study, out of which seventy three were excluded since they did not have malignancy. We used a 22GUS needle Mediglobe^®^ (called simple needle 22G), a modified 22GUB Mediglobe^®^ (called modified needle 22G) and a 21GUS needle Olympus^®^. The different shapes and designs of the needles can be viewed in **Figure 1.** We used the needles as follows; first patient simple needle, second patient modified needle and third patient 21G needle. The simple 22G Mediglobe^®^ and 21G Olympus^®^ have exactly the same shape.

**Figure 2:**
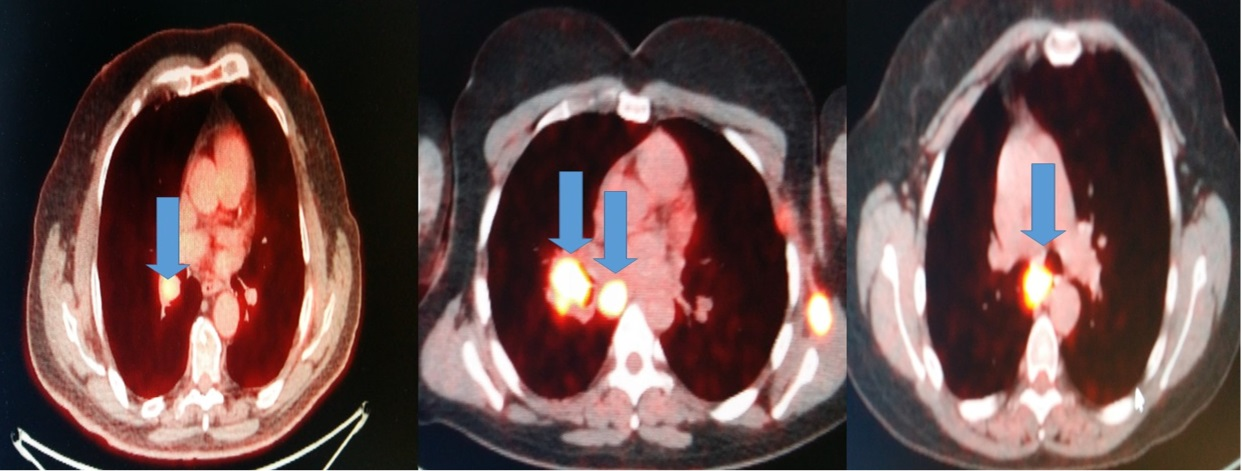
Blue arrows indicate the positive lymphnodes that were punctured (SUV>3).

All patients had before the EBUS-TBNA PET-CT performed. We made four punctures to the most positive lymphnode according to each PET-CT and a cell block was created from each lymphnode **Figure 2**.

In 23 cases we punctured two lymphnodes and two different cell blocks were obtained one from each lymphnode station. These patients were again excluded from the main results. A convex-probe EBUS PENTAX EB-1970UK was used to take biopsies from all patients. All patients were sedated and a rigd bronchoscope (STORZ 12 mm with a 11 mm working channel) or tracheal tube number 8.5 or 9 was instered. The mean procedure time from intubation to last biopsy (four in total for every site) was 15 minutes. In the operating theater there was the operator, a nurse and the anesthisiologist. In all patients jet-ventilation respiration mode was used in order to avoid hypercapnia 16. In the case of the 21G needle Olympus^®^, this needle can be used in the PENTAX convex endoscope, however; it does not lock, and therefore it must be grasped to the connection site. The patients included were either Stage IIIb or Stage IV. All punctured lymphnode varied in a diameter range from 1-3 cm. We tried different lymphnode stations in order to asses the elasticity of the new modifed 22GUB Mediglobe^®^. Limitations of our study was inability to use 19G needle, since our EBUS-TBNA system is the order version (2010 purchase) this means that we can only load 21G and 22G needles. The newer endoscopes (2019) can load up to 19G needles. Moreover; all EBUS-TBNA convex endoscopes have the limitation that when using the 19G the operator cannot take biopsies from small lesions ≤2 cm because of the risk of severe adverse effects, such pneumothorax and hemothorax. Therefore we can use the 19G needle only in large ≥3 cm lesion far away from vessels. However, this should be another study evolving other types of cancer such as lymphoma because 19G is mostly used when there is a suspicion of such case 17. However; we should mention that in the study by Lim CE et al. 17 and Elmufdi FS et al. 18 when the 22G was compared to 19G needle the results were similar for the diagnosis of lymphoma. Therefore, we should always keep in mind that the EBUS operator must have a pathology department with sufficient experience.

### Cell-Blocks

**Figure 3:**
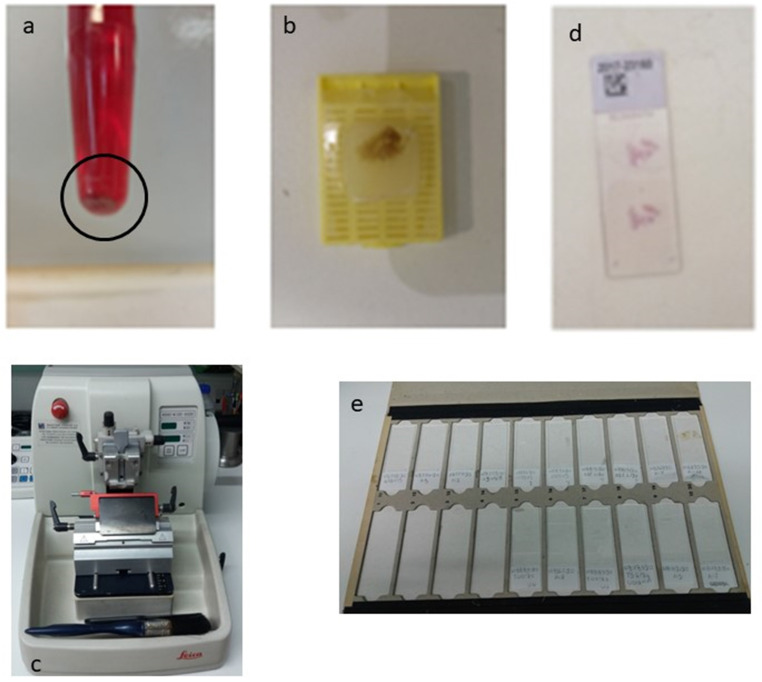
The procedure of the cell block required multiple successive centrifugations of the cellular material, of the fine needle aspiration and the appearance of a precipitate at the bottom of the eppendorf (**a**). Then carefully remove the overlying suspension, preferably using a pipette, and then collect the underlying solid material. This is placed on a cassette that has a filter at its base, for the safest shielding of the material during processing The cassette follows the process of graduated over-night dehydration and then embedded in paraffin and this is the “cell block” (**b**). Three micron tissue sections were taken for histological examination in a semi-automated microtome (**c**), after Haematoxylin/Eosin (H/E) stained for the morphologic study of the material, or unpainted slides for their use in immunohistochemical or molecular techniques (**d, e**).

The speciment of fine needle aspiration (FNA) remains in the hub of cytolyt solution until it arrives in the laboratory. The material was centrifuged in a centrifuge tube at 2000 rpm for 10 minutes. Microscopic tissue fragments are easily recoverable in paraffin cellblock and it follows the protocol of a tissue device in the histopathology laboratory. This process duration was 10 h 41 min. Routine H&E staining is used on all cellblock sections. Each glass slice had tissue sections of 2μ (**Figure 3**).

## Results

### Statistical analysis

All categorical data were tabulated to find frequency distributions.

To detect any beneficial effect of the innovative needle 2 (as compared to others) in joint with the possible effects of position and size of lymph nodes on the number of slices created, a three-way analysis of variance (fixed effects) was employed. Tukey's pairwise differences between means were employed wherever needed. Normality and homogeneity of standardized residuals was also tested. The 0.05 level of statistical significance was considered as a reference probability value.

**Table 1** shows the distribution of node categories as exemplified by their position, size and number of slices formation. It is noteworthy the equal distribution of patients among the three needles whereas the node size 2-3 cm comprises nearly 50% of the study. Lymph nodes are equally partitioned among positions 3 to 6 and halving in positions 7 and 8. Rare occurrence is noted in position 2. Nearly 30% of 9 slices results from all the techniques applied in the study.

**Figure 4:**
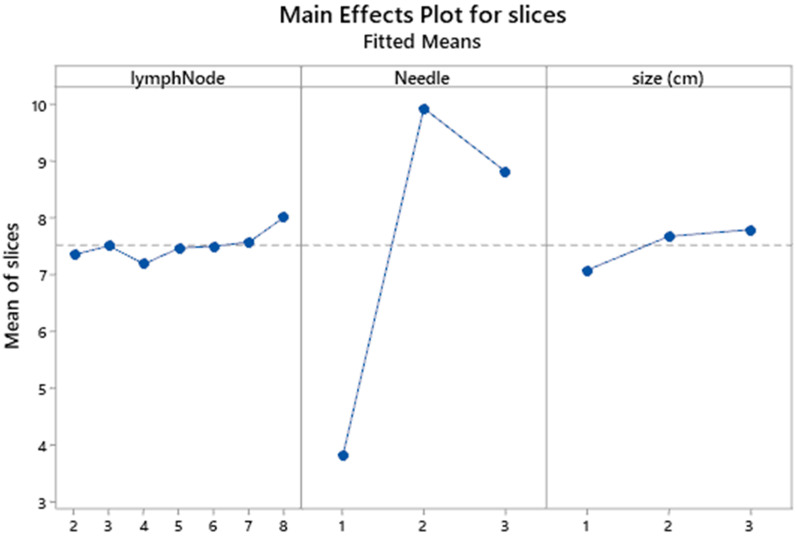
Main effects plot of lymph position and size and needle performance. lymphnode 1 is lympnode station 7; 2 is lympnode station 4R; 3 is lympnode station 4L; 4 is lympnode station 11s; 5 is lympnode station 11i; 6 is lympnode station 11L; 7 is lympnode station 10R; 8 is lympnode station 10L; 9 is biopsy from 2 lympnode stations. Size cm: 1 ≥1; 2 ≥2; 3 ≥3.

The general linear model of slices versus lymph node position and size and needle performance including their interaction effects is shown in **Table 2.** Obviously the linear effects contribute 82.6% of the total variation in which the needle effect dominates with 81.23%, obscuring thereby the effects of lymph position and size. That particular performance is best illustrated in the main effects plot (**Figure 4**) where a large scale distance of responses is generated for the number of slices among needles and also a neglected effect of size differences.

**Figure 5:**
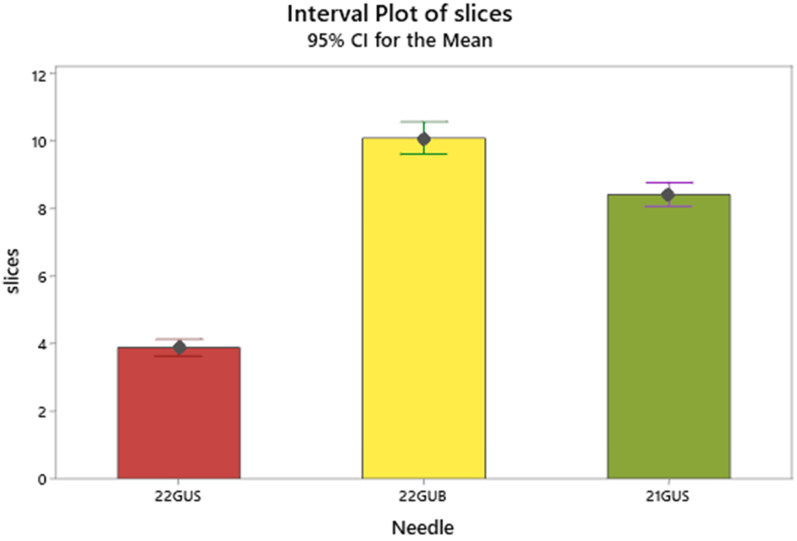
Interval plot of mean slices with the 95% confidence intervals. Intervals that do not overlap denote significant differences of means.

The needle effect is better clarified by the Tukey's comparison of means (**Table 2 and Figure 5**) in which needle 2 exhibits a superior number of mean slices formation (9.9), arrayed next but closely by the needle 3 (8.8) and very distantly by the needle 1 (3.8).

**Figure 6:**
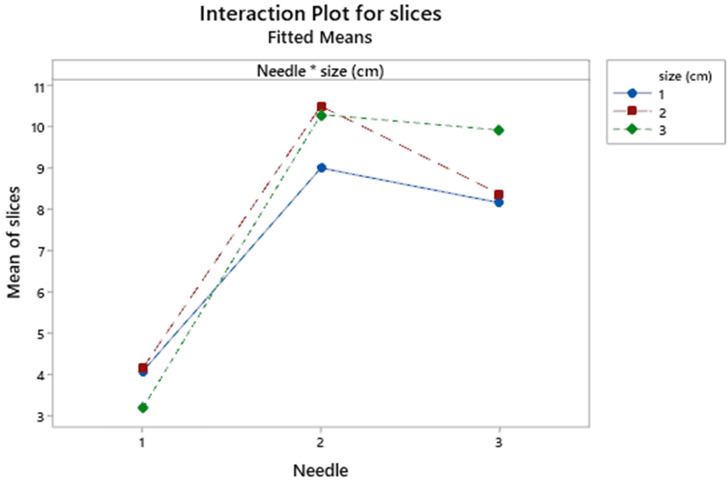
Interaction plot for needle and size effects. Size cm: 1 ≥1; 2 ≥2; 3 ≥3.

Needle and node size interact significantly but loosely (*p*=0.036) showing merely a lower number of slices for size 2-3 in the needle 3 against needle 2 (**Figure 6**).

## Discussion

Endobronchial ultrasound convex probe was mainly designed to access lymphnode stations of the mediastinum for lung cancer staging and for diagnosing non visible central lesions. During the years that this technique is being used several diagnosis were made 19. The main needle that is being used is the 22G needle. There are studies that indicate that 21G needle is more efficient with less false negative cancer results or sarcoidosis results 20. There is also the 25G needle, however; the sample is only cytological. 22G needle is more efficient when compared to 25G needle 21. The 25G needle can be used by the former convex probe EBUS endoscope or the new thin convex probe EBUS which can access additional lymphnode stations or more peripheral lesions 22, 23. There is also the 19G that has been used by the esophageal ultrasound endoscopic system (EUS) 24. The 19G needle is supposed to have the less false negative percentage for lymphoma; however, this is not true 25, 26. We can diagnose B-, T- Non-Hodgkin lymphoma and other hematological malignancies from cell blocks (22G needles) 19, 27. In order to diagnose Hodgkin lymphoma we need larger tissue samples with specific architecture, which is not possible with smaller samples such as 22G and 21G 17. However, in several cases this is not possible even with 19G needles. In the study by Elmufdi FS et al. 18, there was no difference in the overall diagnostic yield between 19 and 21 G needles. Further studies are needed to confirm the trend of the superiority of 19 G in cancerous lymph node. A very important factor that has to be considered is the experience of the pathology lab that is going to handle the biopsy sample. Therefore EBUS-TBNA samples indifferent of the needle size have to be handled by pathology labs with relevant experience in this type of sample 28. Another issue is that 19G needles are more rigid and therefore several lymphnode stations, specifically those of a size ≤1 cm in diameter are not easily accessible. Moreover; those lymphnodes that are both ≤1 cm and close to large vessels are considered a contraindication for this large diameter needle due to the possible adverse effects. Novel 19G needles with higher flexibility are on the way 29, 30. In any case all needles 19G, 22G and 21G have enough material for molecular evaluation 2, 31. It is clear from our study that the architecture of the tip of the needle plays a crucial role and therefore we should focus also on this issue along with the expert pathology evaluation of the sample. The 22GUB modified needle equally efficient with the 21GUS needle for non-small cell lung cancer, metastatic disease from prostate, gastrointestinal tract, breast cancer, and non-Hodgkin lymphoma. We demonstrated a method for the evaluation of the sample size with glass slices produced from the cell blocks. In the future we need to have common methods for evaluation of sample tools.

## Figures and Tables

**Table 1:** Frequency distribution of the variables under study

Variable	Count	Percentage (%)
**lymphNode**		
2	5	3.88
3	24	18.60
4	23	17.83
5	27	20.93
6	26	20.16
7	13	10.08
8	11	8.53
***Totals***		
***N***	**129**	
*	**23**	
**Needle**		
1	42	32.56
2	44	34.11
3	43	33.33
***Totals***		
***N***	**129**	
*	**23**	
**Size (cm)**		
1	39	30.47
2	60	46.88
3	29	22.66
***Totals***		
***N***	**128**	
*	**24**	
**Slices**		
3	16	12.40
4	15	11.63
5	11	8.53
7	15	11.63
8	7	5.43
9	38	29.46
10	9	6.98

lymphnode 1 is lympnode station 7; 2 is lympnode station 4R; 3 is lympnode station 4L; 4 is lympnode station 11s; 5 is lympnode station 11i; 6 is lympnode station 11L; 7 is lympnode station 10R; 8 is lympnode station 10L; 9 is biopsy from 2 lympnode stations. Size(cm): 1 ≥1; 2 ≥2; 3 ≥3.

**Table 2:** A three-way analysis of variance including the percentage contribution of linear and interaction effects

Factor		Type		Level		Values	
lymphNode		Fixed		7		2-8	
Needle		Fixed		3		1-3	
Size (cm)		Fixed		3		1-3	
**Analysis of Variance (ANOVA)**							
***Source***	***DF***	***Seq SS***	***Contribution (%)***	***Adj SS***	***Adj MS***	***Fvalue***	***Pvalue***
lymphNode	6	11.84	1.12	4.993	0.832	0.58	0.744
Needle	2	859.28	81.23	613.369	306.685	214.65	0.000
Size (cm)	2	5.53	0.52	9.817	4.908	3.44	0.036
lymphNode*Needle	12	9.03	0.85	10.818	0.902	0.63	0.811
Needle*size (cm)	4	27.82	2.63	27.818	6.954	4.87	0.001
**Error**	**101**	**144.30**	**13.64**	**144.303**	**1.429**		
**Total**	**127**	**1057.80**	**100.00**				

lymphnode 1 is lympnode station 7; 2 is lympnode station 4R; 3 is lympnode station 4L; 4 is lympnode station 11s; 5 is lympnode station 11i; 6 is lympnode station 11L; 7 is lympnode station 10R; 8 is lympnode station 10L; 9 is biopsy from 2 lympnode stations. Size(cm): 1 ≥1; 2 ≥2; 3 ≥3.

**Table 3:** Grouping information using the Tukey method of pairwise mean-comparisons

Needle	*N*	mean	grouping
2	44	9.93541	A
3	43	8.82072	B
1	41	3.79726	C

Means that do not share a letter are significantly different.
